# External beam radiotherapy for prostate cancer: What are the current research trends and hotspots?

**DOI:** 10.1002/cam4.3700

**Published:** 2021-01-21

**Authors:** Rui Li, Xia Liu, Bo Yang, Jie Qiu

**Affiliations:** ^1^ Department of Radiation Oncology Peking Union Medical College Hospital Peking Union Medical College Chinese Academy of Medical Sciences Beijing China; ^2^ Graduate School of Peking Union Medical College Beijing China

**Keywords:** EBRT, global trends, prostate cancer, scientometric analysis, visualization analysis

## Abstract

**Background:**

The external beam radiotherapy (EBRT) applied for prostate cancer (PCa) has been one of the most important and hottest research fields over recent decades. This study aimed to explore the research hotspots of EBRT in PCa and help the researchers have a clear and intuitive reference basis for later researches.

**Methods:**

The literature scientometric analysis related to “EBRT applied for PCa” was conducted via the Web of Science Core Collection from 2010 to 2019. The Microsoft Office Excel 2019 and CiteSpace V. 5.7.R1 software were introduced for visualizing and analyzing the data.

**Results:**

A total of 7860 relevant papers were extracted and downloaded. A total of 7828 papers were extracted and analyzed after data cleansing by CiteSpace. The tendency of published papers was comprehensively increasing from 2010 to 2019. Among all 73 countries/regions, USA published the most papers, accounting for 39%, which was the most active contributor with most publications. Australia (Centrality: 0.18), England (Centrality: 0.12) were cooperating most cohesively with other countries. Univ Toronto was the most productive institute (229), while Harvard Univ (Centrality: 0.67) had extensive collaborations with other institutes. The International journal of Radiation Oncology Biology Physics had the largest number of publications and the highest number of co‐citations. Briganti A had the largest volume of publications. D'Amico AV had the highest number of co‐citations. Four latest and largest clusters were identified as oligometastases, salvage therapy (SRT), prostate‐specific membrane antigen (PSMA), and hypofractionation. Thirteen references became strongest burst citations lasting until 2019. The studies of “oligometastases,” “SRT,” “PSMA,” “hypofractionation,” “postoperative radiotherapy,” and “dose and fraction regimen changes” were prevailing in the recent years.

**Conclusion:**

The “oligometastases,” “SRT,” “PSMA,” “hypofractionation,” “postoperative radiotherapy,” and “dose and fraction regimen changes” may be the state‐of‐art research frontiers, and related studies will advance in this field over time.

## Graphical abstract

We forecast the research tendency in the field of EBRT applied for prostate cancer: The “oligometastases”, “SRT”, “PSMA”, “hypofractionation”, “postoperative radiotherapy” and “dose and fraction regimen changes” may be the state‐of‐art research frontiers, and related studies would advance in this field over time.

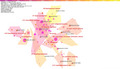

## 1 INTRODUCTION

Prostate cancer (PCa) has become the third main cause of cancer‐induced deaths, with the most common internal malignancy affecting sufferers.^1^ Radiation therapy is an effective treatment option for PCa patients,^2^ which is applicable either as a method of external beam radiation therapy (EBRT) or brachytherapy.^3^ Many therapeutic strategies for EBRT in PCa could be selected as required of accurate and superior management, allowing for the high‐dose delivery to increase the probability of disease control with a lower occurrence of adverse effects.^4^ The alternative types for EBRT in PCa are non‐dose‐escalation conventionally fractionated radiotherapy (non‐DE‐CFRT), DE‐CFRT, hypofractionated radiotherapy (HFRT), stereotactic body radiotherapy (SBRT), etc.^3^

Several studies have highlighted the current status and progress in the field of EBRT in PCa.^3^, ^5^, ^6^, ^7^, ^8^ However, there are few papers using CiteSpace for mining data in the field of radiotherapy. Up to our best knowledge, this study is the early research that applying CiteSpace visualizes and better understands the landscape of global research trends and hotspots from big data of radiotherapy. It may be necessary for promoting the research agenda.

In this study, the bibliometrics and visualization tools are used for providing an objective and comprehensive summary of the research status and hotspots in this filed to help the researchers fully grasp the application status of EBRT in PCa and give a clear and intuitive reference basis for later researches.

## 2 METHODS

### 2.1 Database sources and search strategy

A comprehensive search strategy was conducted to identify the publications in EBRT applied for PCa, which was comprised by three groups of terms (PCa, radiotherapy/EBRT, and brachytherapy terms groups) in the title from the Web of Science Core Collection (WoSCC). Then, the Boolean operation (Appendix S1) was used to combine the two terms group (PCa and radiotherapy/EBRT), and excluded the related studies of brachytherapy terms group, with multi‐checks to confirm the correlation between the results and search terms.

**FIGURE 1:**
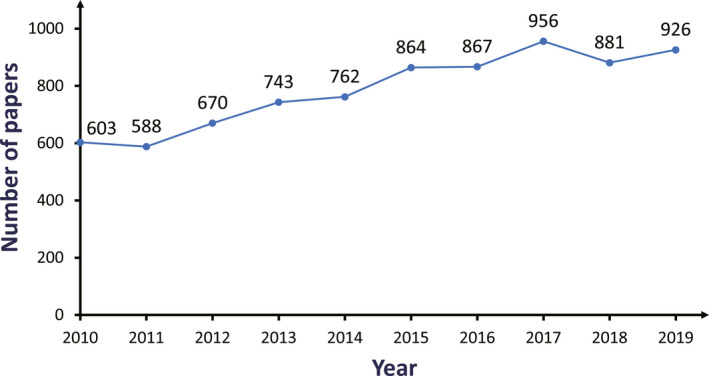
Number of papers on EBRT for PCa area from 2010 to 2019

**FIGURE 2:**
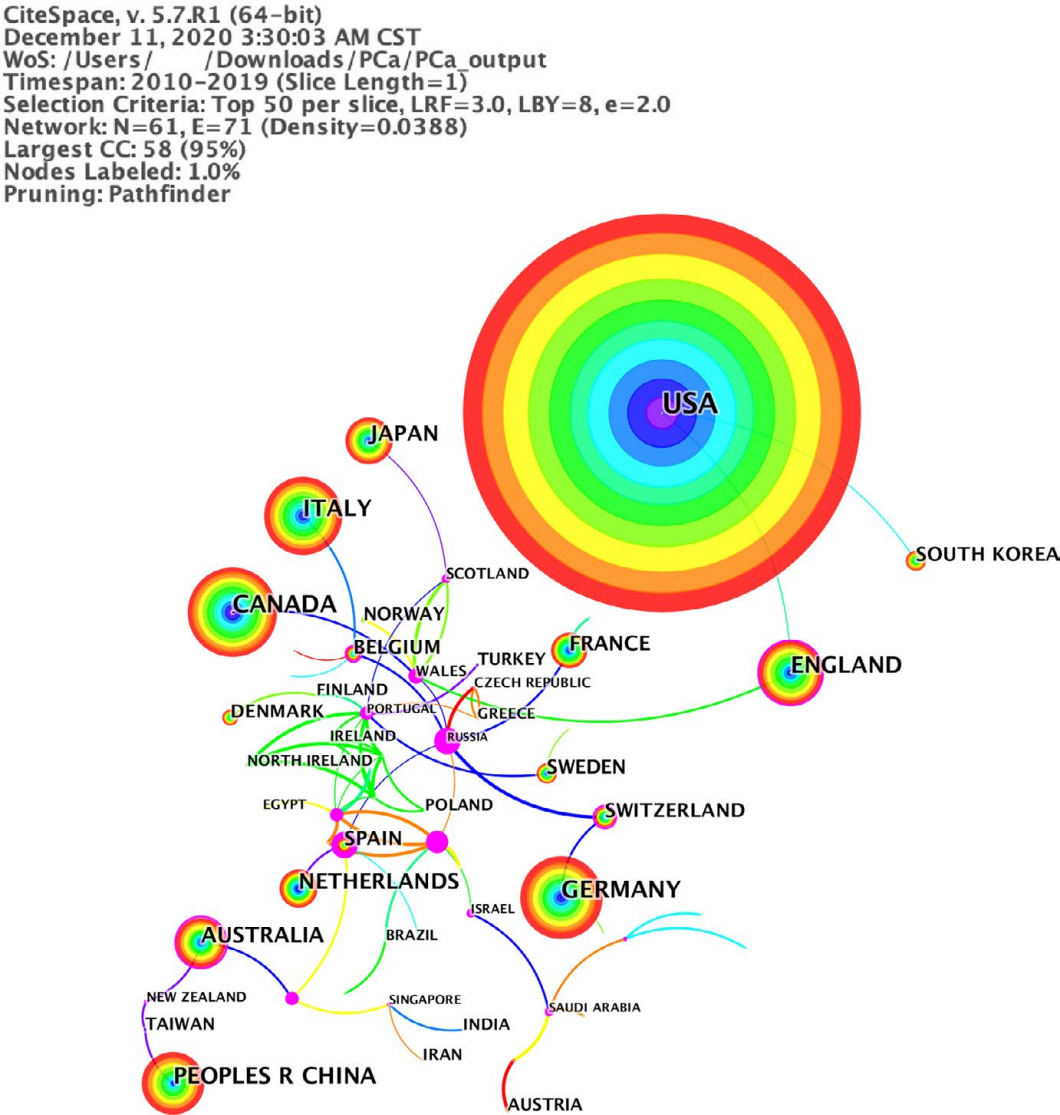
A visualization of the country collaboration network

The WoSCC covered one of the largest worldwide databases of peer‐reviewed publications,^9^, ^10^ including SCI‐EXPANDED, A&HCI, SSCI, BKCI‐S, etc., which was ubiquitously applied in the bibliometric research. The timeframe for this search was set from 2010 to 2019, and papers were extracted from this period (Figure 1). Detailed search strategies could be found in Appendix S1. The document type was limited to Article (Figure 2), and the search language was restricted to English. Finally, 7860 results were ascertained in this study.

### 2.2 Data analysis and visualization

All 7860 papers were extracted with full records and cited references in the WoSCC, the retrieval results were exported to both Microsoft Office Excel 2019 and plain texts for analyzing. The Microsoft Office Excel 2019 was used for analyzing the distribution of publication types and the trend of the numbers of annual publications. The CiteSpace V. 5.7.R1 software^11^, ^12^ was utilized for knowledge mapping and bibliometrics investigations through the plain texts.

## 3 RESULTS

### 3.1 Characteristics of worldwide papers on EBRT in PCa

In total, 7860 articles from 2010 to 2019 were retrieved and analyzed. As shown in Figure 1, the tendency of published papers was comprehensively increasing from 2010 to 2019. These articles were authored by 30 377 authors from 7034 institutes, which were published in 1139 journals, with contributions from 73 countries/regions.

After importing data to CiteSpace V. 5.7.R1 software for removing duplications and data cleansing, zero duplication was found, and the total 7828 papers were extracted and analyzed through data cleansing.

### 3.2 Countries co‐operation network on EBRT in PCa

All 7860 papers were published in 73 countries/regions (WoSCC). Figure 2 presented a network of collaborating countries from 2010 to 2019, with the minimum of two papers. Details of the top 10 countries with the largest number of papers are presented in Table S1. Nine countries of the top 10 productive countries were from the developed countries except China. These 10 countries totally published 7846 papers, accounting for nearly 100% of the total 7828 papers (CiteSpace). Many articles in these 10 countries might be published by multinational cooperation. USA covered about 39% (3071 papers) of the total 7828 papers, which was four times than that of Canada (733 papers). The centrality was also called betweenness centrality. The higher centrality value one node (country, institutes, etc.) had, the more active, stronger, or closer role it could play in the cooperation relationship with other nodes. The centrality of western countries (such as Australia (0.18), England (0.12)) was high. China (528 papers) and Japan (437 papers) were the only two Asian country that entered the top 10 productive countries with the lowest centrality values (0).

### 3.3 Institutes co‐operation network on EBRT in PCa

**FIGURE 3:**
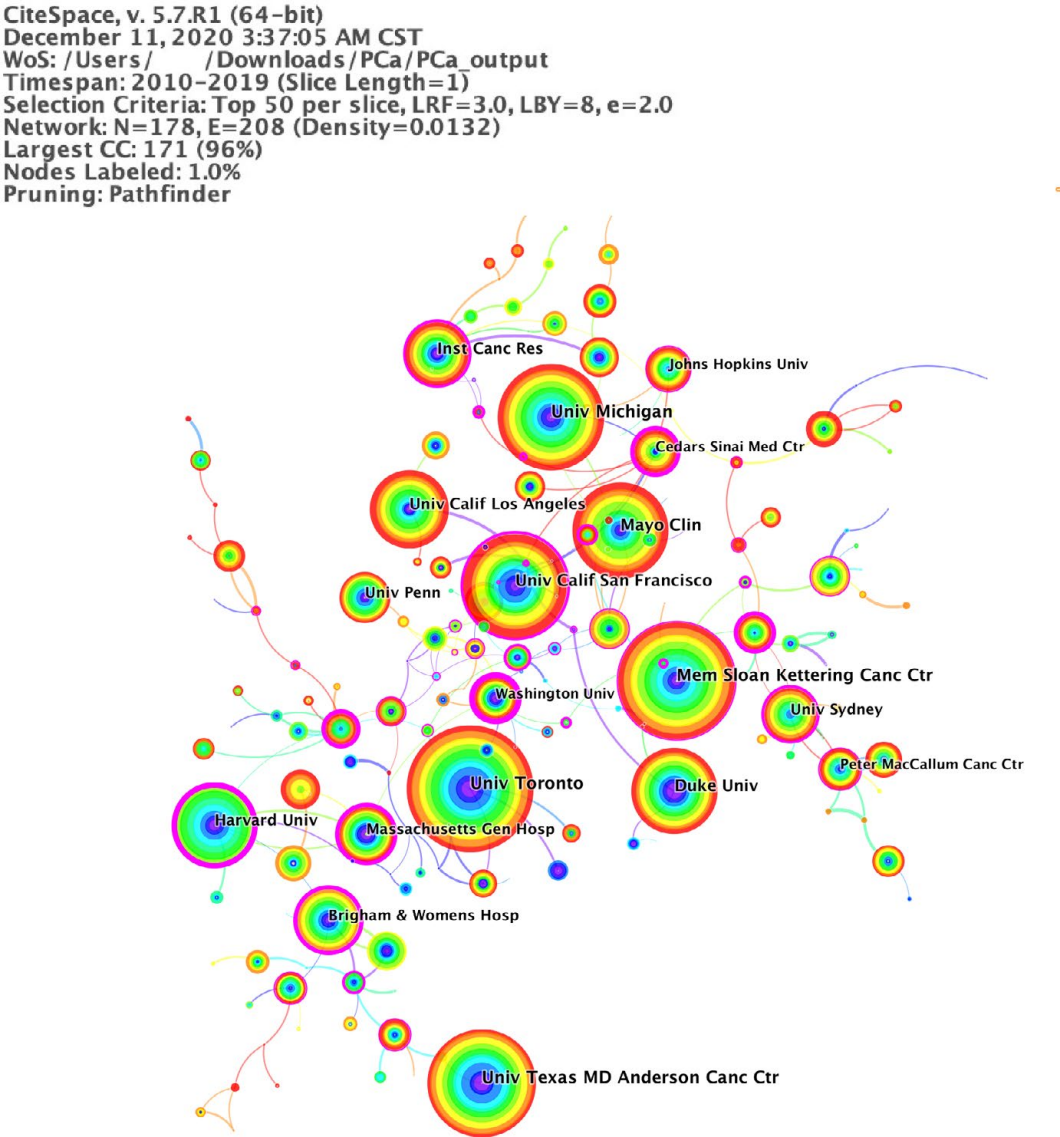
A visualization of the institute collaboration network

All 7860 papers were published in 7034 institutes. Figure 3 showed the network of collaboration institutes from 2010 to 2019, with the minimum of four outputs. The top 10 high output institutes are shown in Table S2, which made up of about 22% of the total 7828 outputs. The top 10 research institutes with most publications were all derived from developed countries, with nine institutes from USA, one institute from Canada. Univ Toronto was the largest productive institute with 229 outputs, followed by Mem Sloan Kettering Canc Ctr (211 outputs), Univ Texas MD Anderson Canc Ctr (194 outputs). Harvard Univ (0.67) had the highest centrality.

### 3.4 Journal co‐citation network on EBRT in PCa

**FIGURE 4:**
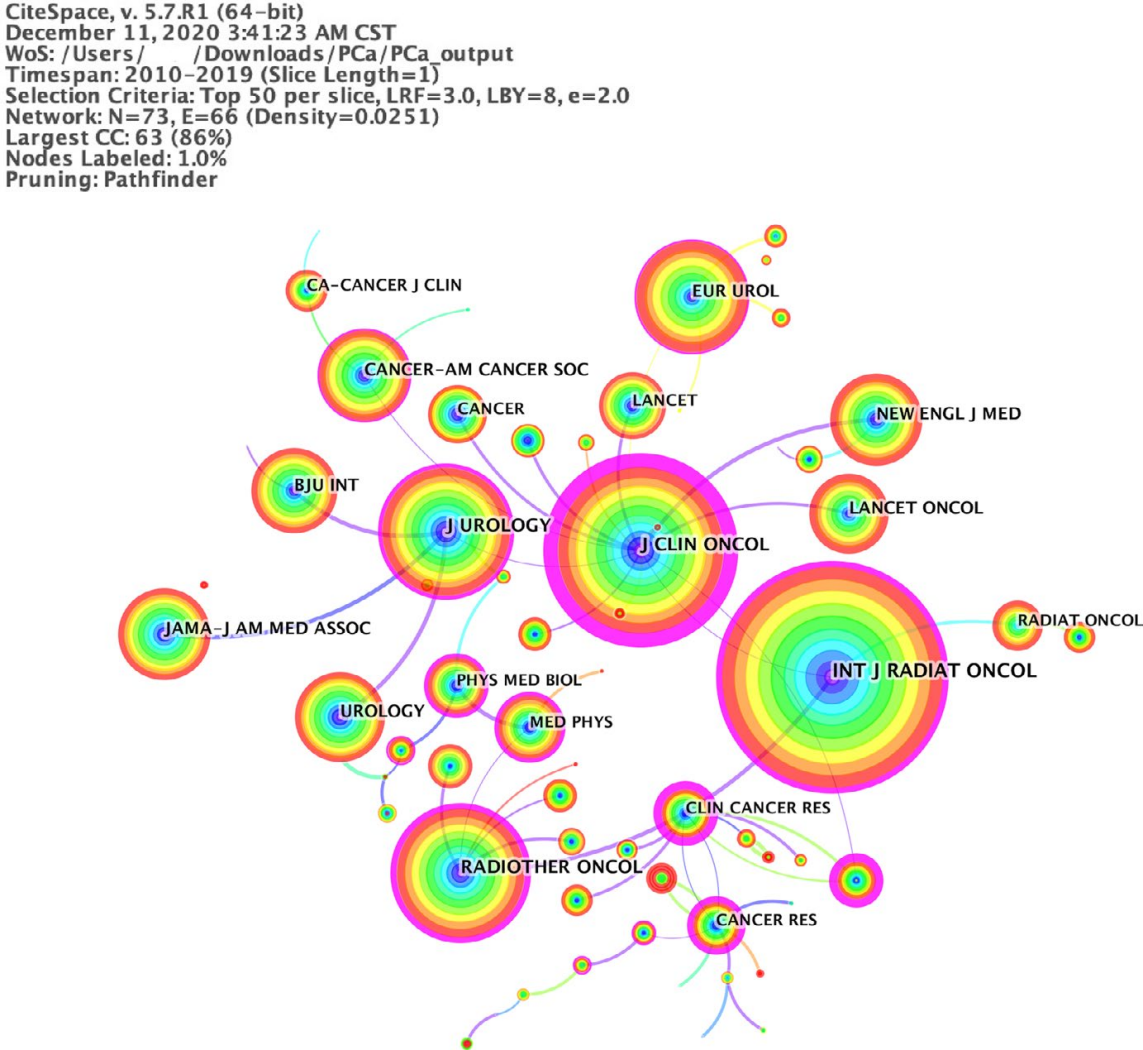
A visualization of the journal co‐citation network

A total of 7860 papers were published across 1139 journals, 27% of the total papers published by the top 10 journals with high outputs. Among the top 10 journals with high outputs (Table S3), the International journal of Radiation Oncology Biology Physics published the most papers (IF‐2019: 5.859; 522 papers, 6.6%), followed by Radiotherapy and Oncology (IF‐2019: 4.856; 5.252; 271 papers, 3.4%). Figure 4 presented the journal co‐citation network from 2010 to 2019, with the minimum of 32 co‐citations. The top 10 most frequently cited journals are shown in Table S4. The International journal of Radiation Oncology Biology Physics was the most prominent journal with 5451 co‐citations, which had a profound influence on correlated studies in this field, followed by journal of Clinical Oncology (4229 co‐citations) and Radiotherapy and Oncology (3313).

### 3.5 Author co‐citation network on EBRT in PCa

**FIGURE 5:**
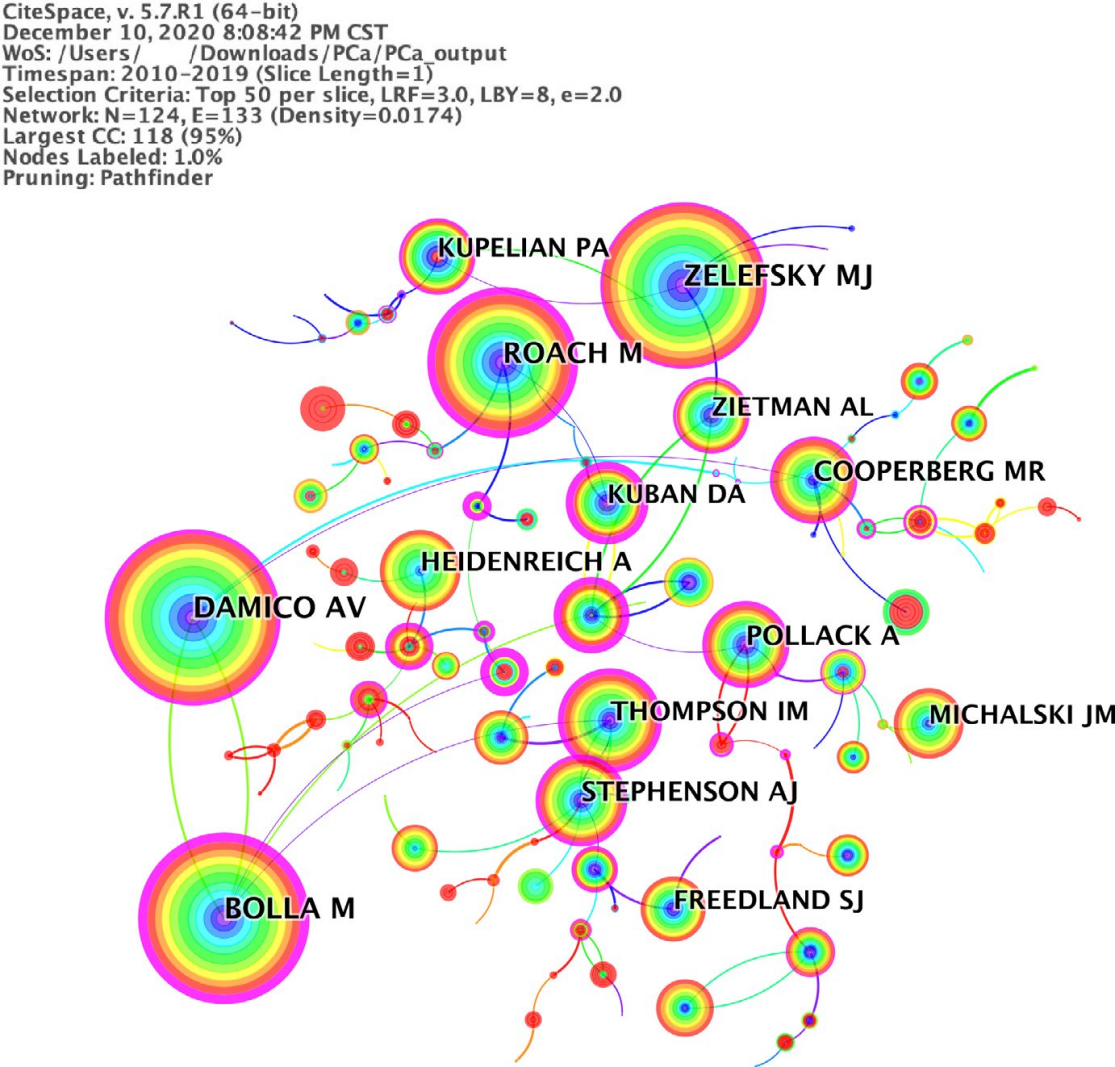
A visualization of the author co‐citation network

A total of 7860 papers were totally published by 30 377 authors, with 9% of the total papers published by the top 10 productive authors. Briganti A ranked the first (103 papers; 1.3%) among the top 10 productive authors, followed by Nguyen PL (86 papers; 1.1%), and Karnes RJ (85 papers; 1.1%) (Table S5). Each of the top 10 authors contributed at least 51 papers. Figure 5 showed the network of author co‐citation from 2010 to 2019, with the minimum of 19 co‐citations. The Table S6 presented the top 10 most frequently cited authors. D'Amico AV was the most prominent author with 1,136 co‐citations, followed by Zelefsky MJ (1067) and Bolla M (1029).

### 3.6 Research hotspots

#### 3.6.1 Paper co‐citation network on EBRT in PCa

**FIGURE 6:**
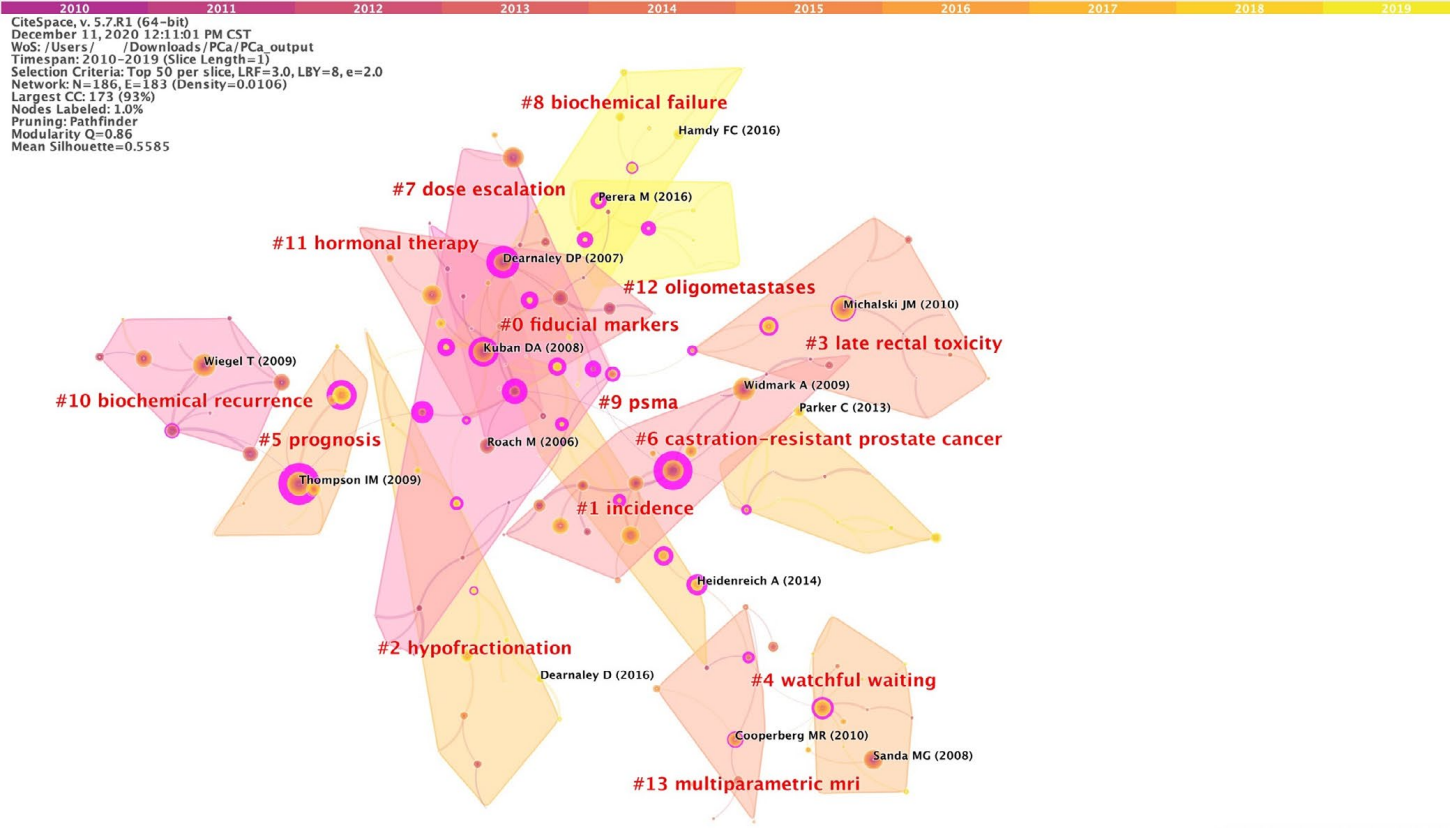
A visualization of the paper co‐citation network

All papers from 2010 to 2019 were loaded into CiteSpace V. 5.7.R1 software for analyzing the paper co‐citation network, and the time slice was selected for 1 year with pathfinder. Figure 6 showed the paper co‐citation network, with 186 nodes, 183 links, and 14 main clusters, which were generated with the Modularity Q of 0.86 and Mean Silhouette of 0.5585. The nodes and links were displayed the cited references and co‐citation relationships from the extracted papers, respectively. The link colors directly reflected the time slice, with colder colors representing early years, warmer colors representing late years. The 14 main clusters were generated by CiteSpace V. 5.7.R1 software and the clusters were marked by utilizing the keyword terms and a log‐likelihood ratio weighting algorithm, which was used for calculating and determining each type of label by presenting the core concept of each cluster with given professional words.

### 3.7 Citation data with the top four latest co‐citation clusters

The top four latest clusters (oligometastases, salvage therapy (SRT), prostate‐specific membrane antigen (PSMA), and hypofractionation) are presented in Table S7. The typical five papers^13^, ^14^, ^15^, ^16^, ^17^ in the cluster oligometastases were mainly about the early detection and treatment for PCa oligometastases^13^ which could improve clinical effects by identifying patients who might be benefit from local therapy rather than palliative care.^14^, ^15^, ^16^, ^17^ The typical five papers^18^, ^19^, ^20^, ^21^, ^22^ in the cluster SRT majorly studied that the application of SRT might increase clinical treatment results when implemented with the lower prostate‐specific antigen (PSA) level at initiation,^19^, ^20^, ^21^ higher radiation dose,^19^, ^20^ and combined with systematic management like androgen deprivation therapy (ADT). Five representative papers^23^, ^24^, ^25^, ^26^, ^27^ in the cluster PSMA suggested that PSMA was a promising new tool in the diagnosis of restaging,^23^ recurrence,^25^, ^26^, ^27^ and metastasis^24^ of PCa. The papers^28^, ^29^, ^30^, ^31^, ^32^ in the hypofractionation cluster were majorly about the moderately HFRT was efficacious either in the adjuvant/salvage setting of postoperative PCa. The results could be seen in Tables S8–S11, respectively.

#### 3.7.1 Citation bursts on EBRT in PCa

The citation bursts were considered as papers that were received drastic augments in the references, could partially response the research dynamics and hotspots of a field. The whole papers were exported by CiteSpace V. 5.7.R1 software each year from 2010 to 2019, with a total 58 references tagged as citation bursts (Table S12), which highlighted the hotspots and tendency of this field during this period. Thirteen references^33^, ^34^, ^35^, ^36^, ^37^, ^38^, ^39^, ^40^, ^41^, ^42^, ^43^, ^44^, ^45^ became strongest burst citations lasting until 2019 (1), which reflected the latest and freshest hotspots in this field. Nine papers^33^, ^34^, ^35^, ^36^, ^37^, ^38^, ^39^, ^40^, ^41^ were well classified into two aspects of hotspots besides four papers.^42^, ^43^, ^44^, ^45^ The 1st,^38^ 3th,^39^ 5th,^40^ and 12th^41^ burst citations were both about the topic of “postoperative radiotherapy”; the 4nd,^33^ 7rd,^36^ 8th,^35^ 9th,^34^ and 11th^37^ were mainly focused on “dose and fraction regimen changes.”

**TABLE 1:** 13 References with the Strongest Citation Bursts lasting until 2019

Rank	References	Year	Strength	Begin	End	2010–2019
1	Heidenreich A, 2014, EUR UROL, V65, P467, DOI	2014	**29.5678**	2015	2019	
2	Sweeney CJ, 2015, NEW ENGL J MED, V373, P737, DOI	2015	**24.8281**	2016	2019	
3	Wiegel T, 2014, EUR UROL, V66, P243, DOI	2014	**21.8722**	2016	2019	
4	Miralbell Raymond, 2012, INT J RADIAT ONCOL BIOL PHYS, V82, P0, DOI	2012	**20.5106**	2016	2019	
5	Bolla M, 2012, LANCET, V380, P2018, DOI	2012	**20.5018**	2014	2019	
6	Afshar‐Oromieh A, 2014, EUR J NUCL MED MOL I, V41, P11, DOI	2014	**20.2421**	2016	2019	
7	King CR, 2012, INT J RADIAT ONCOL, V82, P877, DOI	2012	**19.0749**	2016	2019	
8	Pollack A, 2013, J CLIN ONCOL, V31, P3860, DOI	2013	**18.9734**	2015	2019	
9	Dearnaley DP, 2014, LANCET ONCOL, V15, P464, DOI	2014	**15.5711**	2015	2019	
10	Resnick MJ, 2013, NEW ENGL J MED, V368, P436, DOI	2013	**14.8902**	2016	2019	
11	Zelefsky MJ, 2012, INT J RADIAT ONCOL, V84, P125, DOI	2012	**13.241**	2014	2019	
12	Thompson IM, 2013, J UROLOGY, V190, P441, DOI	2013	**10.6127**	2016	2019	
13	Parker C, 2013, NEW ENGL J MED, V369, P213, DOI	2013	**8.9275**	2016	2019	

## 4 DISCUSSION

### 4.1 General data

The field of EBRT for PCa has been obtained great attention for decades, and the total papers are increasing over time. Nine countries of the top 10 productive countries are from developed countries except China. Cooperation among countries is extensively close, especially in the western countries. In particular, the value of centrality of Australia is the highest, which may indicate that it has an obvious advantage over other countries in a certain research direction, resulting in a high degree of cooperation with other countries. China and Japan are the only two Asian countries that have entered the top 10 productive countries, but their cooperation intensities are weaker than those of European, North American, and Oceanian countries. The research institutes of this field are also keeping close cooperation with each other. The top 10 research institutes with most publications are all derived from developed countries, with nine institutes from USA, one institute from Canada.

### 4.2 Citation data

Among all the 1139 academic journals, the International Journal of Radiation Oncology Biology Physics ranks first concerning the number of published papers. And the number of citations of this journal also ranks first, showing the profound influence in this field. Each of the top 10 authors contributes at least 51 papers. As a result, they are classified as “prolific authors.” D'Amico AV, Zelefsky MJ, and Bolla M are the most prominent authors with high co‐citations, indicating that the three authors have high‐quality papers.

### 4.3 Co‐citation cluster hotspots

#### 4.3.1 The management of oligometastases

The early detection for PCa oligometastases^13^ could improve clinical effects by identifying patients who might be benefit from local treatment rather than palliative care.^14^, ^15^, ^16^, ^17^ ^68^Ga‐PSMA‐11 PET/CT (positron emission tomography/computed tomography) has been exhibited superior results for the early detection of oligometastases, especially at low PSA levels.^13^, ^46^ Recently, there are three main approaches for the effective management of oligometastatic PCa: locally radical therapy;^15^ targeted approach like surgery or SBRT for metastases;^15^, ^16^, ^17^ systematic management by chemotherapy or ADT for occult diseases.^15^ SBRT provides high rates of local control^16^, ^17^ and prolonged progression‐free survival (PFS)^17^ for patients with oligometastatic PCa, which should be taken evaluation for curation or delaying systemic therapy.^16^

#### 4.3.2 The application of SRT

Ghadjar et al^18^ suggested that SRT when combined with ADT could increase overall survival (OS) for patients with pre‐SRT PSA values of ≥0.7 ng/ml.^18^ However, the implementation time of SRT may affect the clinical treatment results of PCa patients.^19^ Christopher et al^20^ assumed that early SRT (ESRT) might be equivalent to adjuvant radiotherapy (ART) if SRT is implemented with the lower PSA level at initiation and higher radiation dose.^20^ Ost et al^19^ further demonstrated this perspective that ESRT (PSA ≤ 0.5) displayed similar 3‐year biochemical relapse‐free survival (bRFS) to ART (PSA < 0.2) (ART: 92% vs. ESRT: 86%, *p* = .67) and superior outcome than late SRT (LSRT) (PSA > 0.5) (ESRT: 86% vs. LSRT: 46%, *p* < .001) for high‐risk PCa patinets using high‐dose radiotherapy.^19^ But Hwang et al^21^ indicated that the clinical outcomes of ESRT (PSA: 0.2–0.4) were still significantly lower when compared to ART (PSA < 0.1) for high‐risk patients, like freedom from biochemical failure and OS,^21^ which might be due to the lack of PSA sensitivity when biochemical recurrence, thus, leading the poor results of SRT.^19^ To improve bRFS, Kashihara et al^22^ recommended that the adequate inclusion of the seminal vesicle bed (SVB) was necessary when performing SRT, especially for patients with positive margins at the base of the prostate.^22^

#### 4.3.3 The detection by PSMA

^68^Ga‐PSMA‐11 PET/CT was significantly more sensitive than standard imaging (e.g., bone scan or CT)^23^ and other PET/CT tracers^47^ for PCa patients and was highly consistent among radiation oncologists with high‐experience levels for PCa staging, especially for detecting the lymph nodes or bone lesions.^48^ Furthermore, the intervention of ^68^Ga‐PSMA‐PET can detect the recurrent PCa lesions at the serum PSA levels low enough to cause changes in the stage of primary TNM, which may alter the treatment regimen,^24^, ^49^ target delineation^50^, ^51^ for routine SRT,^51^ and give better detection of tumor recurrence or metastasis in radiotherapy management. Mazzola et al^25^ supported that ^68^Ga‐PSMA could be used for detecting early biochemical recurrence setting, which allowed the early identification of potential metastatic lesions and provided a trustworthy method for pre‐SRT staging.^25^ Calais et al^26^ also found that 19% PCa patients could be observed at least one PSMA positive lesion that were not contained by the consensus clinical target volume (CTV) areas that delineated by CT,^26^ even made a major impact on SRT planning when corrected the CTVs by using the ^68^Ga‐PSMA PET.^26^ Albisinni et al^27^ suggested that ^68^Ga‐PSMA PET/CT might make modifications of the first proposed treatment strategy in about 99/131 (76%) men, what predominantly included continuing surveillance, SRT, SBRT, hormonal manipulations, etc.^27^

#### 4.3.4 The implementation of hypofractionation

Moderately HFRT for PCa is well tolerated and feasible in the localized^52^, ^53^ or locally advanced, high‐risk or N1 PCa patients, and possesses low severe late toxicity rates during short‐^54^/long‐term follow‐up.^52^ Cuccia et al^55^ also concluded that moderately HFRT was efficacious either in the adjuvant/salvage setting of postoperative PCa, with reports of excellent rates of biochemical control, promising results in relapse‐free survival^30^ and reducing the overall treatment time.

### 4.4 Citation burst hotspots

#### 4.4.1 Postoperative radiotherapy

Even though the tumors are removed completely by radical prostatectomy, it still remains controversial whether radiotherapy of postoperative patients with PCa is required. Although not all PSA relapsing will change to clinical progression,^38^, ^56^ recurrence rate is still uncertain, especially in the intermediate‐risk or high‐risk patients. ART was offered by radiation oncologists for PCa patients with obvious pathologic findings at prostatectomy, while SRT was provided for patients with PSA or local recurrence after prostatectomy and no evidence of distant metastasis.^41^ Wiegel et al^39^ demonstrated that ART had an advantage in 10‐year PFS compared to wait‐and‐see policy (56% vs. 35%, *p* < .0001). However, there may be overtreating for patients never experience relapse by implementing with ART^57^ or undertreating for patients with microscopic metastases after surgery by implementing with SRT.^19^ Some studies^40^, ^41^ also suggested that not all patients would benefit from ART, postoperative irradiation needed take the age,^40^ patient's history, preferences and tolerance, functional status, quality of life, short‐/long‐term side effects of radiotherapy, etc. into consideration.^41^ For example, the more excess mortality was observed in patients over 70 years/older treated by ART when compared with those who only used wait‐and‐see policy after surgery (42.6% vs. 19.6%).^40^

#### 4.4.2 Dose and fraction regimen changes

The dose range of the non‐DE‐CFRT is limited to 64–70 Gy, due to the long‐term toxicity risks of threatening the rectum and bladder.^4^ With the enhancement of imaging‐guided techniques and advanced linac systems,^37^ the DE‐CFRT could reach doses up to 74–80 Gy in 2 Gy per fraction with improved freedom from biochemical and clinical progression,^34^ however, this advantage might not change into the improvement of OS.^34^ The efficacy data of escalated‐dose treatment should be balanced with the increments of acute and late toxicities, which also emphasizes the utilization of appropriate modern radiotherapy technology to decrease side effects.^34^ Miralbell et al^33^ proposed that the α/β ratio for prostate tumors was potentially lower than that for late toxicity, which alluded that utilizing fewer numbers and larger sizes of fractions were anticipated. Hence, the fractionated sensitivity differential between tumor and normal tissue contributed HFRT and SBRT schedules to preferred alternatives for PCa administration, which was also very beneficial logistically in limited‐resource settings.^33^, ^35^, ^36^ It is also noteworthy that some PCa patients with compromised urinary function may become worse after HFRT, which should not be the ideal candidates of this treatment.^35^

#### 4.4.3 Other possible hotspots

Proton therapy and heavy ion therapy have the characteristics of Bragg peak, which contribute a higher dose ratio between the target and adjacent normal tissue compared with photon therapy. Studies have shown that PCa patients are acceptable by treating with post‐prostatectomy proton therapy, which has the favorable acute gastrointestinal and genitourinary toxicity rates through the minimum 3 months follow‐up.^58^ Magnetic resonance imaging (MRI) is often used in EBRT for PCa because of its better soft tissue recognition, and the generation and use of MRI‐linacs make it possible for PCa adaptive radiotherapy.^59^ Future daily plan adaptation will permit the reduction of target margin on the MRI‐linac and also potentially further decrease the dose of organs at risk.^60^ Therefore, proton, heavy ions, and MRI‐linac have also become potential research hotspots.

The strategy to administer radiotherapy in PCa patients, therefore, needs to be carried out by the patient and multidisciplinary treatment group with full consideration of a series of patient/clinical relate factors (e.g., age/basic PSA level/dose and fraction regimen/ functional status) to achieve better prognosis.

## 5 CONCLUSION

This scientometric investigation on the application of EBRT for PCa is helpful for researchers to grasp the hotspots and trends of this field. By retrieving and collecting documents from the WoSCC, the data analysis is more objective, comprehensive, and reproducible, which can offer large information in this field, even helping researchers obtain massive data in a short time.

However, there are also some limitations in this study. First of all, the WoSCC is the only data source of this study, some databases, like PubMed, Scopus, and Google Scholar, are not involved and analyzed. Second, the majority of papers are published in English and the few non‐English papers are not included. Third, a deviation may exist in the results of study measurement analysis and the actual status of the studies, because the cited times of some papers published recently is not high. Forth, there may be some missing items in the plain texts downloaded from WoSCC, which makes the total results of CiteSpace 7828, while WoSCC is 7860. But the missing papers were only 32, which might not affect the total results of this study. Finally, all papers were retrieved on 20 August 2020, but the literatures published in 2020 were not included in this analysis. Nevertheless, this study covered an overwhelming majority in the documents published since the year of 2010. The small number of papers may not change the entire tendency in this study.

In conclusion, the “oligometastases,” “SRT,” “PSMA,” “hypofractionation,” “postoperative radiotherapy,” and “dose and fraction regimen changes” may be the hottest research frontiers in radiotherapy field. The four clusters and 13 references with strongest burst citations should be considered carefully for the fresh researchers in this field. This study will be of great significance in the field of EBRT for PCa, particularly for clinical decision‐making and management of PCa patients.

## CONFLICT OF INTEREST

The authors made no competing interests.

## AUTHOR CONTRIBUTIONS

RL conducted the search strategy, analyzed the data, wrote and revised the manuscript. XL and BY conceived and designed the study, critically revised the manuscript. JQ critically revised the manuscript and provided the final approval of the manuscript. All authors read and approved the final manuscript.

## ETHICAL STATEMENT

The authors are accountable for all aspects of the work in ensuring that questions related to the accuracy or integrity of any part of the work are appropriately investigated and resolved.

## INFORMED CONSENT

This study is a bibliometrics analysis, which does not contain any studies with human or animals performed by any of the authors.

## AVAILABILITY OF DATA AND MATERIAL

Data and material can be obtained upon request.

## Supporting information

Table S1‐S12

Appendix S1
