# Supplementary material for: External beam radiotherapy for prostate cancer: What are the current research trends and hotspots?
Source: Cancer Med. 2021 Jan 21;10(2):772–82. doi: 10.1002/cam4.3700 (PMC7877352; doi:10.1002/cam4.3700)
Supplement: Supplementary file 1 — Table S1‐S12 [file CAM4-10-772-s001.docx]

**Supplementary Tables:**

**S. Tab. 1.** Top 10 countries based on count.

**S. Tab. 2.** Top 10 institutes based on count.

**S. Tab. 3.** Top 10 most productive journals.

**S. Tab. 4.** Top 10 journals with most co-citation count.

**S. Tab. 5.** Top 10 most productive authors.

**S. Tab. 6.** Top 10 authors with most co-citation count.

**S. Tab. 7.** Details of the 14 clusters.

**S. Tab. 8.** The representative papers of Cluster 12.

**S. Tab. 9.** The representative papers of Cluster 8.

**S. Tab. 10.** The representative papers of Cluster 9.

**S. Tab. 11.** The representative papers of Cluster 2.

**S. Tab. 12.** Top 58 References with the Strongest Citation Bursts.

**S. Tab. 1.** Top 10 countries based on count.

| Rank | Count | Centrality | Country |
| --- | --- | --- | --- |
| 1 | 3,071 | 0.06 | USA |
| 2 | 733 | 0 | CANADA |
| 3 | 699 | 0.06 | GERMANY |
| 4 | 656 | 0 | ITALY |
| 5 | 569 | 0.12 | ENGLAND |
| 6 | 528 | 0 | PEOPLES R CHINA |
| 7 | 444 | 0.18 | AUSTRALIA |
| 8 | 437 | 0 | JAPAN |
| 9 | 376 | 0 | NETHERLANDS |
| 10 | 333 | 0.06 | FRANCE |

**S. Tab. 2.** Top 10 institutes based on count.

| Rank | Count | Centrality | Institutes |
| --- | --- | --- | --- |
| 1 | 229 | 0.09 | Univ Toronto |
| 2 | 211 | 0.18 | Mem Sloan Kettering Canc Ctr |
| 3 | 194 | 0.02 | Univ Texas MD Anderson Canc Ctr |
| 4 | 192 | 0.07 | Univ Michigan |
| 5 | 188 | 0.21 | Univ Calif San Francisco |
| 6 | 175 | 0.04 | Mayo Clin |
| 7 | 160 | 0.09 | Duke Univ |
| 8 | 144 | 0.04 | Univ Calif Los Angeles |
| 9 | 134 | 0.67 | Harvard Univ |
| 10 | 116 | 0.25 | Inst Canc Res |

**S. Tab. 3.** Top 10 most productive journals.

| Rank | Journals | Count | % of 7,860 | IF (2019) |
| --- | --- | --- | --- | --- |
| 1 | INTERNATIONAL JOURNAL OF RADIATION ONCOLOGY BIOLOGY PHYSICS | 522 | 6.641 | 5.859 |
| 2 | RADIOTHERAPY AND ONCOLOGY | 271 | 3.448 | 4.856 |
| 3 | MEDICAL PHYSICS | 231 | 2.939 | 3.317 |
| 4 | RADIATION ONCOLOGY | 225 | 2.863 | 2.817 |
| 5 | BJU INTERNATIONAL | 190 | 2.417 | 4.806 |
| 6 | PHYSICS IN MEDICINE AND BIOLOGY | 176 | 2.239 | 2.883 |
| 7 | EUROPEAN UROLOGY | 153 | 1.947 | 5.925 |
| 8 | STRAHLENTHERAPIE UND ONKOLOGIE | 124 | 1.578 | 2.899 |
| 9 | JOURNAL OF UROLOGY | 122 | 1.552 | 5.925 |
| 10 | JOURNAL OF APPLIED CLINICAL MEDICAL PHYSICS | 120 | 1.527 | 1.679 |

**S. Tab. 4.** Top 10 journals with most co-citation count.

| Rank | Count | Centrality | Cited Journals | IF (2019) |
| --- | --- | --- | --- | --- |
| 1 | 5,451 | 0.6 | INTERNATIONAL JOURNAL OF RADIATION ONCOLOGY BIOLOGY PHYSICS | 5.859 |
| 2 | 4,229 | 1.2 | JOURNAL OF CLINICAL ONCOLOGY | 32.956 |
| 3 | 3,313 | 0.53 | RADIOTHERAPY AND ONCOLOGY | 4.856 |
| 4 | 3,311 | 0.23 | JOURNAL OF UROLOGY | 5.925 |
| 5 | 2,822 | 0.14 | EUTOPEAN UROLOGY | 17.947 |
| 6 | 2,480 | 0.14 | CANCER-AM CANCER SOC | 5.742 |
| 7 | 2,436 | 0 | UROLOGY | 1.924 |
| 8 | 2,397 | 0 | JAMA-JOURNAL OF THE AMERICAN MEDICAL ASSOCIATION | 45.540 |
| 9 | 2,378 | 0.09 | NEW ENGLAND JOURNAL OF MEDICINE | 74.699 |
| 10 | 2,243 | 0.05 | BJU INTERNATIONAL | 4.806 |

**S. Tab. 5.** The top 10 most productive authors.

| Rank | Author | Count | % of 7860 |
| --- | --- | --- | --- |
| 1 | BRIGANTI A | 103 | 1.310 |
| 2 | NGUYEN PL | 86 | 1.094 |
| 3 | KARNES RJ | 85 | 1.081 |
| 4 | MONTORSI F | 83 | 1.056 |
| 5 | FENG FY | 73 | 0.929 |
| 6 | D'AMICO AV | 59 | 0.751 |
| 7 | OST P | 56 | 0.712 |
| 8 | SPRATT DE | 53 | 0.674 |
| 9 | FREEDLAND SJ | 52 | 0.662 |
| 10 | GANDAGLIA G | 51 | 0.649 |

**S. Tab. 6.** Top 10 authors with most co-citation count.

| Rank | Count | Centrality | Cited Authors |
| --- | --- | --- | --- |
| 1 | 1,136 | 0.54 | D'AMICO AV |
| 2 | 1,067 | 0.32 | Zelefsky MJ |
| 3 | 1,029 | 1.03 | Bolla M |
| 4 | 905 | 0.94 | Roach M |
| 5 | 632 | 0.61 | Thompson IM |
| 6 | 573 | 0.53 | Stephenson AJ |
| 7 | 570 | 0.06 | Heidenreich A |
| 8 | 567 | 0.34 | Cooperberg MR |
| 9 | 554 | 0.52 | Pollack A |
| 10 | 507 | 0.34 | Zietman AL |

**S. Tab. 7.** Details of the 14 clusters.

| Cluster ID | Size | Silhouette | Mean（Year） | Label (LLR) |
| --- | --- | --- | --- | --- |
| 0 | 16 | 0.827 | 2007 | fiducial markers (15.49, 1.0E-4); organ motion (11.14, 0.001); tracking (10.31, 0.005); image-guided radiotherapy (10.2, 0.005); intensity-modulated radiotherapy (7.34, 0.01) |
| 1 | 16 | 0.972 | 2009 | incidence (10.93, 0.001); androgen-deprivation therapy (6.5, 0.05); intermediate risk (6.5, 0.05); risk groups (5.66, 0.05); pc tumor progression (5.46, 0.05) |
| 2 | 15 | 1 | 2012 | hypofractionation (27.78, 1.0E-4); cyberknife (22.99, 1.0E-4); stereotactic body radiotherapy (20.63, 1.0E-4); toxicity (12.35, 0.001); stereotactic body radiation therapy (11.46, 0.001) |
| 3 | 13 | 1 | 2009 | late rectal toxicity (23.74, 1.0E-4); vmat (18.3, 1.0E-4); ntcp (15.78, 1.0E-4); prostate radiotherapy (15.78, 1.0E-4); plan comparison (11.56, 0.001) |
| 4 | 13 | 1 | 2012 | watchful waiting (19.81, 1.0E-4); gleason grade (9.88, 0.005); comparative effectiveness research (9.88, 0.005); unfavorable intermediate risk (9.88, 0.005); neoplasm grading (9.88, 0.005) |
| 5 | 13 | 0.969 | 2011 | prognosis (16.95, 1.0E-4); salvage radiotherapy (10.15, 0.005); adverse pathologic features (9.86, 0.005); adjuvant (8.06, 0.005); biochemical recurrence (5.1, 0.05) |
| 6 | 13 | 0.992 | 2012 | castration-resistant prostate cancer (24.98, 1.0E-4); immunotherapy (20.33, 1.0E-4); docetaxel (15.23, 1.0E-4); androgen receptor (10.89, 0.001); radium-223 (10.14, 0.005) |
| 7 | 12 | 0.935 | 2008 | dose escalation (17.54, 1.0E-4); tumor delineation (10.54, 0.005); igrt (5.3, 0.05); protons (5.26, 0.05); prostate margin (5.26, 0.05) |
| 8 | 12 | 0.959 | 2016 | biochemical failure (19.55, 1.0E-4); salvage therapy (10.53, 0.005); postprostatectomy (6.12, 0.05); salvage radiation (6.12, 0.05); relative survival (4.87, 0.05) |
| 9 | 11 | 0.962 | 2014 | psma (19.05, 1.0E-4); ct (14.36, 0.001); ga-68-psma (14.36, 0.001); pet/ct (14.35, 0.001); recurrence (13.29, 0.001) |
| 10 | 11 | 1 | 2006 | biochemical recurrence (11.44, 0.001); salvage (8.67, 0.005); adjuvant radiation (7.16, 0.01); risk stratification (6.63, 0.05); bone scan (6.25, 0.05) |
| 11 | 10 | 0.951 | 2005 | hormonal therapy (8.29, 0.005); androgen deprivation (7.85, 0.01); bicalutamide (5.97, 0.05); timing of hormonal therapy (5.62, 0.05); neoadjuvant hormone therapy (5.62, 0.05) |
| 12 | 10 | 0.984 | 2016 | oligometastases (21.99, 1.0E-4); oligometastatic prostate cancer (17.11, 1.0E-4); oligometastasis (12.1, 0.001); metastasis-directed therapy (10.96, 0.001); sbrt (10.27, 0.005) |
| 13 | 8 | 0.97 | 2011 | multiparametric mri (15.68, 1.0E-4); bombesin (10.44, 0.005); diagnosis (6.77, 0.01); infrafractional motion (5.21, 0.05); serum-free medium culture (5.21, 0.05) |

**S. Tab. 8.** The representative papers of Cluster 12.

| Serial Number | Title |
| --- | --- |
| 1 | Imaging modalities in synchronous oligometastatic prostate cancer |
| 2 | Oligometastases in genitourinary tumors: Recent insights and future molecular diagnostic approach |
| 3 | The role of local therapy for oligometastatic prostate cancer: Should we expect a cure? Urologic clinics of north America |
| 4 | Oligometastases from prostate cancer: local treatment with stereotactic body radiotherapy (SBRT) |
| 5 | Progression-free survival following stereotactic body radiotherapy for oligometastatic prostate cancer treatment-naïve recurrence: |

**S. Tab. 9.** The representative papers of Cluster 8.

| Serial Number | Title |
| --- | --- |
| 1 | Use of androgen deprivation and salvage radiation therapy for patients with prostate cancer and biochemical recurrence after prostatectomy |
| 2 | A matched control analysis of adjuvant and salvage high-dose postoperative intensity-modulated radiotherapy for prostate cancer |
| 3 | The timing of salvage radiotherapy after radical prostatectomy: a systematic review |
| 4 | Comparison between adjuvant and early-salvage postprostatectomy radiotherapy for prostate cancer with adverse pathological features |
| 5 | Importance of the site of positive surgical margin in salvage external beam radiation therapy for biochemical recurrence of prostate cancer after radical prostatectomy |

**S. Tab. 10.** The representative papers of Cluster 9.

| Serial Number | Title |
| --- | --- |
| 1 | A multicenter prospective clinical trial of ^68^Gallium PSMA HBED-CC PET-CT restaging in biochemically relapsed prostate carcinoma: oligometastatic rate and distribution compared with standard imaging |
| 2 | ^68^Ga-PSMA-PET for radiation treatment planning in prostate cancer recurrences after surgery: Individualized medicine or new standard in salvage treatment |
| 3 | New metabolic tracers for detectable PSA levels in the post-prostatectomy setting: is the era of melting glaciers upcoming? |
| 4 | Ga-68-PSMA-11 PET/CT mapping of prostate cancer biochemical recurrence after radical prostatectomy in 270 patients with a PSA level of less than 1.0 ng/ml: impact on salvage radiotherapy planning |
| 5 | Clinical impact of ^68^Ga-prostate-specific membrane antigen (PSMA) positron emission tomography/computed tomography (PET/CT) in patients with prostate cancer with rising prostate-specific antigen after treatment with curative intent: preliminary analysis of a multidisciplinary approach |

**S. Tab. 11.** The representative papers of Cluster 2.

| Serial Number | Title |
| --- | --- |
| 1 | Hypofractionated helical 3DCRT and IMRT systems for the post-operative treatment of prostate cancer: a mono-institutional report |
| 2 | Hypofractionation with VMAT versus 3DCRT in post-operative patients with prostate cancer |
| 3 | Clinical results of a moderately hypofractionated radiotherapy in postoperative prostate carcinoma |
| 4 | Toxicity and biochemical outcomes of hypofractionated intensity modulated post-operative radiation therapy for prostate cancer |
| 5 | Postoperative hypofractionated radiotherapy for prostate cancer |

**S. Tab. 12.** Top 58 References with the Strongest Citation Bursts.

| Rank | References | Year | Strength | Begin | End | 2010 - 2019 |
| --- | --- | --- | --- | --- | --- | --- |
| 1 | [Roach M, 2006, INT J RADIAT ONCOL, V65, P965, DOI](http://dx.doi.org/10.1016%2Fj.ijrobp.2006.04.029) | 2006 | **48.0785** | 2010 | 2014 | ▃▃▃▃▃▂▂▂▂▂ |
| 2 | [Zietman AL, 2005, JAMA-J AM MED ASSOC, V294, P1233, DOI](http://dx.doi.org/10.1001%2Fjama.294.10.1233) | 2005 | **45.962** | 2010 | 2013 | ▃▃▃▃▂▂▂▂▂▂ |
| 3 | [Bolla M, 2005, LANCET, V366, P572, DOI](http://dx.doi.org/10.1016%2FS0140-6736%2805%2967101-2) | 2005 | **45.6459** | 2010 | 2013 | ▃▃▃▃▂▂▂▂▂▂ |
| 4 | [Heidenreich A, 2014, EUR UROL, V65, P124, DOI](http://dx.doi.org/10.1016%2Fj.eururo.2013.09.046) | 2014 | **35.3467** | 2015 | 2017 | ▂▂▂▂▂▃▃▃▂▂ |
| 5 | [Peeters STH, 2006, J CLIN ONCOL, V24, P1990, DOI](http://dx.doi.org/10.1200%2FJCO.2005.05.2530) | 2006 | **32.6505** | 2010 | 2014 | ▃▃▃▃▃▂▂▂▂▂ |
| 6 | [Heidenreich A, 2011, EUR UROL, V59, P61, DOI](http://dx.doi.org/10.1016%2Fj.eururo.2010.10.039) | 2011 | **31.2441** | 2012 | 2014 | ▂▂▃▃▃▂▂▂▂▂ |
| 7 | [Heidenreich A, 2014, EUR UROL, V65, P467, DOI](http://dx.doi.org/10.1016%2Fj.eururo.2013.11.002) | 2014 | **29.5678** | 2015 | 2019 | ▂▂▂▂▂▃▃▃▃▃ |
| 8 | [Siegel R, 2014, CA-CANCER J CLIN, V64, P9, DOI](http://dx.doi.org/10.3322%2Fcaac.21208) | 2014 | **28.6203** | 2014 | 2016 | ▂▂▂▂▃▃▃▂▂▂ |
| 9 | [Jemal A, 2010, CA-CANCER J CLIN, V60, P277, DOI](http://dx.doi.org/10.3322%2Fcaac.20073) | 2010 | **28.5624** | 2011 | 2013 | ▂▃▃▃▂▂▂▂▂▂ |
| 10 | [Thompson IM, 2006, JAMA-J AM MED ASSOC, V296, P2329, DOI](http://dx.doi.org/10.1001%2Fjama.296.19.2329) | 2006 | **28.2885** | 2010 | 2014 | ▃▃▃▃▃▂▂▂▂▂ |
| 11 | [Freedland SJ, 2005, JAMA-J AM MED ASSOC, V294, P433, DOI](http://dx.doi.org/10.1001%2Fjama.294.4.433) | 2005 | **28.066** | 2010 | 2013 | ▃▃▃▃▂▂▂▂▂▂ |
| 12 | [Siegel R, 2012, CA-CANCER J CLIN, V62, P10, DOI](http://dx.doi.org/10.3322%2Fcaac.20138) | 2012 | **27.6892** | 2013 | 2015 | ▂▂▂▃▃▃▂▂▂▂ |
| 13 | [Pilepich MV, 2005, INT J RADIAT ONCOL, V61, P1285, DOI](http://dx.doi.org/10.1016%2Fj.ijrobp.2004.08.047) | 2005 | **26.508** | 2010 | 2013 | ▃▃▃▃▂▂▂▂▂▂ |
| 14 | [Jemal A, 2009, CA-CANCER J CLIN, V59, P225, DOI](http://dx.doi.org/10.3322%2Fcaac.20006) | 2009 | **25.2211** | 2010 | 2012 | ▃▃▃▂▂▂▂▂▂▂ |
| 15 | [Sweeney CJ, 2015, NEW ENGL J MED, V373, P737, DOI](http://dx.doi.org/10.1056%2FNEJMoa1503747) | 2015 | **24.8281** | 2016 | 2019 | ▂▂▂▂▂▂▃▃▃▃ |
| 16 | [DAmico AV, 2004, JAMA-J AM MED ASSOC, V292, P821, DOI](http://dx.doi.org/10.1001%2Fjama.292.7.821) | 2004 | **23.5557** | 2010 | 2012 | ▃▃▃▂▂▂▂▂▂▂ |
| 17 | [Bill-Axelson A, 2014, NEW ENGL J MED, V370, P932, DOI](http://dx.doi.org/10.1056%2FNEJMoa1311593) | 2014 | **22.9703** | 2015 | 2017 | ▂▂▂▂▂▃▃▃▂▂ |
| 18 | [Dearnaley DP, 2007, LANCET ONCOL, V8, P475, DOI](http://dx.doi.org/10.1016%2FS1470-2045%2807%2970143-2) | 2007 | **22.0954** | 2010 | 2014 | ▃▃▃▃▃▂▂▂▂▂ |
| 19 | [Wiegel T, 2014, EUR UROL, V66, P243, DOI](http://dx.doi.org/10.1016%2Fj.eururo.2014.03.011) | 2014 | **21.8722** | 2016 | 2019 | ▂▂▂▂▂▂▃▃▃▃ |
| 20 | [Keating NL, 2006, J CLIN ONCOL, V24, P4448, DOI](http://dx.doi.org/10.1200%2FJCO.2006.06.2497) | 2006 | **21.845** | 2010 | 2013 | ▃▃▃▃▂▂▂▂▂▂ |
| 21 | [Dearnaley D, 2012, LANCET ONCOL, V13, P43, DOI](http://dx.doi.org/10.1016%2FS1470-2045%2811%2970293-5) | 2012 | **21.5536** | 2013 | 2016 | ▂▂▂▃▃▃▃▂▂▂ |
| 22 | [Michalski JM, 2013, INT J RADIAT ONCOL, V87, P932, DOI](http://dx.doi.org/10.1016%2Fj.ijrobp.2013.07.041) | 2013 | **21.4025** | 2015 | 2017 | ▂▂▂▂▂▃▃▃▂▂ |
| 23 | [Stephenson AJ, 2004, JAMA-J AM MED ASSOC, V291, P1325, DOI](http://dx.doi.org/10.1001%2Fjama.291.11.1325) | 2004 | **20.6451** | 2010 | 2012 | ▃▃▃▂▂▂▂▂▂▂ |
| 24 | [Miralbell Raymond, 2012, INT J RADIAT ONCOL BIOL PHYS, V82, P0, DOI](http://dx.doi.org/10.1016%2Fj.ijrobp.2010.10.075) | 2012 | **20.5106** | 2016 | 2019 | ▂▂▂▂▂▂▃▃▃▃ |
| 25 | [Bolla M, 2012, LANCET, V380, P2018, DOI](http://dx.doi.org/10.1016%2FS0140-6736%2812%2961253-7) | 2012 | **20.5018** | 2014 | 2019 | ▂▂▂▂▃▃▃▃▃▃ |
| 26 | [Afshar-Oromieh A, 2014, EUR J NUCL MED MOL I, V41, P11, DOI](http://dx.doi.org/10.1007%2Fs00259-013-2525-5) | 2014 | **20.2421** | 2016 | 2019 | ▂▂▂▂▂▂▃▃▃▃ |
| 27 | [Otto K, 2008, MED PHYS, V35, P310, DOI](http://dx.doi.org/10.1118%2F1.2818738) | 2008 | **19.7132** | 2011 | 2014 | ▂▃▃▃▃▂▂▂▂▂ |
| 28 | [Kupelian P, 2007, INT J RADIAT ONCOL, V67, P1088, DOI](http://dx.doi.org/10.1016%2Fj.ijrobp.2006.10.026) | 2007 | **19.3649** | 2010 | 2013 | ▃▃▃▃▂▂▂▂▂▂ |
| 29 | [King CR, 2012, INT J RADIAT ONCOL, V82, P877, DOI](http://dx.doi.org/10.1016%2Fj.ijrobp.2010.11.054) | 2012 | **19.0749** | 2016 | 2019 | ▂▂▂▂▂▂▃▃▃▃ |
| 30 | [Pollack A, 2013, J CLIN ONCOL, V31, P3860, DOI](http://dx.doi.org/10.1200%2FJCO.2013.51.1972) | 2013 | **18.9734** | 2015 | 2019 | ▂▂▂▂▂▃▃▃▃▃ |
| 31 | [Kupelian PA, 2007, INT J RADIAT ONCOL, V68, P1424, DOI](http://dx.doi.org/10.1016%2Fj.ijrobp.2007.01.067) | 2007 | **17.6005** | 2012 | 2014 | ▂▂▃▃▃▂▂▂▂▂ |
| 32 | [Mohler J, 2010, J NATL COMPR CANC NE, V8, P162, DOI](http://dx.doi.org/10.6004%2Fjnccn.2010.0012) | 2010 | **17.2442** | 2011 | 2015 | ▂▃▃▃▃▃▂▂▂▂ |
| 33 | [Heidenreich A, 2008, EUR UROL, V53, P68, DOI](http://dx.doi.org/10.1016%2Fj.eururo.2007.09.002) | 2008 | **16.9103** | 2010 | 2012 | ▃▃▃▂▂▂▂▂▂▂ |
| 34 | [Messing EM, 2006, LANCET ONCOL, V7, P472, DOI](http://dx.doi.org/10.1016%2FS1470-2045%2806%2970700-8) | 2006 | **16.8268** | 2010 | 2014 | ▃▃▃▃▃▂▂▂▂▂ |
| 35 | [Wilt TJ, 2012, NEW ENGL J MED, V367, P203, DOI](http://dx.doi.org/10.1056%2FNEJMoa1113162) | 2012 | **16.5957** | 2014 | 2016 | ▂▂▂▂▃▃▃▂▂▂ |
| 36 | [Denham JW, 2005, LANCET ONCOL, V6, P841, DOI](http://dx.doi.org/10.1016%2FS1470-2045%2805%2970348-X) | 2005 | **16.4958** | 2010 | 2012 | ▃▃▃▂▂▂▂▂▂▂ |
| 37 | [Thompson I, 2007, J UROLOGY, V177, P2106, DOI](http://dx.doi.org/10.1016%2Fj.juro.2007.03.003) | 2007 | **16.4681** | 2013 | 2015 | ▂▂▂▃▃▃▂▂▂▂ |
| 38 | [Klotz L, 2010, J CLIN ONCOL, V28, P126, DOI](http://dx.doi.org/10.1200%2FJCO.2009.24.2180) | 2010 | **16.3214** | 2013 | 2015 | ▂▂▂▃▃▃▂▂▂▂ |
| 39 | [Dearnaley DP, 2014, LANCET ONCOL, V15, P464, DOI](http://dx.doi.org/10.1016%2FS1470-2045%2814%2970040-3) | 2014 | **15.5711** | 2015 | 2019 | ▂▂▂▂▂▃▃▃▃▃ |
| 40 | [Zelefsky MJ, 2008, INT J RADIAT ONCOL, V71, P1028, DOI](http://dx.doi.org/10.1016%2Fj.ijrobp.2007.11.066) | 2008 | **15.253** | 2010 | 2012 | ▃▃▃▂▂▂▂▂▂▂ |
| 41 | [Resnick MJ, 2013, NEW ENGL J MED, V368, P436, DOI](http://dx.doi.org/10.1056%2FNEJMoa1209978) | 2013 | **14.8902** | 2016 | 2019 | ▂▂▂▂▂▂▃▃▃▃ |
| 42 | [Sheets NC, 2012, JAMA-J AM MED ASSOC, V307, P1611, DOI](http://dx.doi.org/10.1001%2Fjama.2012.460) | 2012 | **14.7327** | 2013 | 2017 | ▂▂▂▃▃▃▃▃▂▂ |
| 43 | [Stephenson AJ, 2007, J CLIN ONCOL, V25, P2035, DOI](http://dx.doi.org/10.1200%2FJCO.2006.08.9607) | 2007 | **14.2065** | 2010 | 2015 | ▃▃▃▃▃▃▂▂▂▂ |
| 44 | [Warde P, 2011, LANCET, V378, P2104, DOI](http://dx.doi.org/10.1016%2FS0140-6736%2811%2961095-7) | 2011 | **13.9059** | 2014 | 2016 | ▂▂▂▂▃▃▃▂▂▂ |
| 45 | [Al-Mamgani A, 2008, INT J RADIAT ONCOL, V72, P980, DOI](http://dx.doi.org/10.1016%2Fj.ijrobp.2008.02.073) | 2008 | **13.8132** | 2010 | 2012 | ▃▃▃▂▂▂▂▂▂▂ |
| 46 | [Kuban DA, 2008, INT J RADIAT ONCOL, V70, P67, DOI](http://dx.doi.org/10.1016%2Fj.ijrobp.2007.06.054) | 2008 | **13.78** | 2010 | 2012 | ▃▃▃▂▂▂▂▂▂▂ |
| 47 | [Zelefsky MJ, 2012, INT J RADIAT ONCOL, V84, P125, DOI](http://dx.doi.org/10.1016%2Fj.ijrobp.2011.11.047) | 2012 | **13.241** | 2014 | 2019 | ▂▂▂▂▃▃▃▃▃▃ |
| 48 | [Cooperberg MR, 2010, J CLIN ONCOL, V28, P1117, DOI](http://dx.doi.org/10.1200%2FJCO.2009.26.0133) | 2010 | **12.9067** | 2013 | 2016 | ▂▂▂▃▃▃▃▂▂▂ |
| 49 | [Denham JW, 2011, LANCET ONCOL, V12, P451, DOI](http://dx.doi.org/10.1016%2FS1470-2045%2811%2970063-8) | 2011 | **12.6621** | 2015 | 2017 | ▂▂▂▂▂▃▃▃▂▂ |
| 50 | [Thompson IM, 2013, J UROLOGY, V190, P441, DOI](http://dx.doi.org/10.1016%2Fj.juro.2013.05.032) | 2013 | **10.6127** | 2016 | 2019 | ▂▂▂▂▂▂▃▃▃▃ |
| 51 | [Roehl KA, 2004, J UROLOGY, V172, P910, DOI](http://dx.doi.org/10.1097%2F01.ju.0000134888.22332.bb) | 2004 | **9.8781** | 2010 | 2012 | ▃▃▃▂▂▂▂▂▂▂ |
| 52 | [Parker C, 2013, NEW ENGL J MED, V369, P213, DOI](http://dx.doi.org/10.1056%2FNEJMoa1213755) | 2013 | **8.9275** | 2016 | 2019 | ▂▂▂▂▂▂▃▃▃▃ |
| 53 | [Sanda MG, 2008, NEW ENGL J MED, V358, P1250, DOI](http://dx.doi.org/10.1056%2FNEJMoa074311) | 2008 | **8.3823** | 2012 | 2016 | ▂▂▃▃▃▃▃▂▂▂ |
| 54 | [Trock BJ, 2008, JAMA-J AM MED ASSOC, V299, P2760, DOI](http://dx.doi.org/10.1001%2Fjama.299.23.2760) | 2008 | **7.8271** | 2014 | 2016 | ▂▂▂▂▃▃▃▂▂▂ |
| 55 | [Jones CU, 2011, NEW ENGL J MED, V365, P107, DOI](http://dx.doi.org/10.1056%2FNEJMoa1012348) | 2011 | **6.2355** | 2013 | 2016 | ▂▂▂▃▃▃▃▂▂▂ |
| 56 | [Roach M, 2008, J CLIN ONCOL, V26, P585, DOI](http://dx.doi.org/10.1200%2FJCO.2007.13.9881) | 2008 | **5.5895** | 2010 | 2012 | ▃▃▃▂▂▂▂▂▂▂ |
| 57 | [Zelefsky MJ, 2008, INT J RADIAT ONCOL, V70, P1124, DOI](http://dx.doi.org/10.1016%2Fj.ijrobp.2007.11.044) | 2008 | **4.888** | 2012 | 2015 | ▂▂▃▃▃▃▂▂▂▂ |
| 58 | [DAmico AV, 2008, JAMA-J AM MED ASSOC, V299, P289, DOI](http://dx.doi.org/10.1001%2Fjama.299.3.289) | 2008 | **4.0714** | 2012 | 2016 | ▂▂▃▃▃▃▃▂▂▂ |
