# Supplementary material for: External beam radiotherapy for prostate cancer: What are the current research trends and hotspots?
Source: Cancer Med. 2021 Jan 21;10(2):772–82. doi: 10.1002/cam4.3700 (PMC7877352; doi:10.1002/cam4.3700)
Supplement: Supplementary file 2 — Appendix S1 [file CAM4-10-772-s002.docx]

**Search strategy (Aug 20^th^, 2020):**

Select a database: Web of Science Core Collection

Document types: Article

Language: English

Duration: 2010-2019

Citation Indexes: The Science Citation Index Expanded (SCI-EXPANDED), the Social Sciences Citation Index (SSCI), the Arts & Humanities Citation Index (A&HCI), the Conference Proceedings Citation Index-Science (CPCI-S), the Conference Proceedings Citation Index-Social Science & Humanities (CPCI-SSH), the Book Citation Index-Science (BKCI-S), the Book Citation Index-Social Sciences & Humanities (BKCI-SSH), the Emerging Sources Citation Index (ESCI), the Current Chemical Reactions Expanded (CCR-EXPANDED), the Index Chemicus (IC)

Results: 7,860

Boolean operation rules:

((TS=(("prostate cancer") OR (PCa) OR ("prostate tumor") OR ("prostate tumour") OR ("prostate neoplasm") OR ("prostate carcinoma") OR ("prostate gland cancer") OR ("prostate gland tumor") OR ("prostate gland tumour") OR ("prostate gland neoplasm") OR ("prostate gland carcinoma") OR ("cancer of the prostate gland") OR ("tumor of the prostate gland") OR ("tumour of the prostate gland") OR ("neoplasm of the prostate gland") OR ("carcinoma of the prostate gland"))) AND ((TS=(("radiation oncology") OR ("radiation therapy") OR (radiotherapy) OR (“conformal radiation therapy”) OR ("conformal radiotherapy") OR ("external beam radiation therapy") OR ("external beam radiotherapy") OR (EBRT) OR ("intensity-modulated radiation therapy") OR ("intensity modulated radiation therapy") OR ("intensity-modulated radiotherapy") OR ("intensity modulated radiotherapy") OR (IMRT) OR ("stereotacticbody radiotherapy") OR ("neo-adjuvant chemoradiation") OR ("neoadjuvant chemoradiation") OR ("adjuvant chemoradiation") OR ("stereotacticbody radiation therapy") OR (SBRT) OR ("stereotactic radiosurgery") OR ("stereotactic radiotherapy") OR ("stereotactic radiation therapy") OR ("Image Guided Radiotherapy") OR ("Image Guided Radiation therapy") OR (IGRT) OR ("image guided adaptive radiotherapy") OR ("image guided adaptive radiation therapy") OR ("adaptive radiation therapy") OR ("adaptive radiotherapy") OR (IGART) OR (VMAT) OR ("Volumetric Intensity Modulated Arc Therapy") OR ("proton radiotherapy") OR ("proton radiation therapy"))) NOT (TS=(("Radioisotope Brachytherapy") OR ("Curietherapy") OR ("Interstitial Radiotherapy") OR ("Implant Radiotherapy") OR ("high dose rate") OR (HDR) OR ("low dose rate") OR (LDR) OR ("pulsed dose rate") OR (PDR) OR ("medium dose rate") OR (MDR) OR (brachytherapy)))))
